# The hidden cost of ageing: measuring the impact of health expenditure on poverty among households with older adults in India

**DOI:** 10.3389/fpubh.2026.1950585

**Published:** 2026-10-07

**Authors:** Neeraj Soni, Rajabhushan Jagadish Nayak, Meghna Ghosh, Ashish Singh

**Affiliations:** 1 Department of Economic Affairs, Government of India, New Delhi, India; 2 Sri Sathya Sai Institute of Higher Learning (Deemed to be University), Puttaparthi, India; 3 BITS Law School, Kalyan, India; 4 Shailesh J. Mehta School of Management, Mumbai, India

**Keywords:** ageing, India, out-of-pocket health expenditure, poverty, universal insurance coverage

## Abstract

According to the World Bank’s poverty and inequality platform, 11.09% of India’s population lived below the international poverty line of $2.15 (2017 PPP) per day in 2018. Moreover, 48.2% of total health expenditure is financed through out-of-pocket payments (OOPHE), underscoring their role in driving households into poverty. With 31.4% of households reporting at least one older adult (census 2011), this study focuses on the impoverishing effects of OOPHE among older adults-inclusive households. Using nationally representative data from the NSS 75th round: household social consumption – health (2017–2018), we estimate the poverty headcount and poverty gap at the household level, before and after accounting for OOPHE for households with at least one older adult member. We further employ a binary logistic regression to identify socio-economic and demographic determinants of post-OOPHE impoverishment. The results show that longer hospitalization spells, larger household size, caste status, and reliance on private healthcare significantly elevate the risk of falling into poverty. In contrast, health insurance coverage and higher educational attainment reduce this risk. Despite multiple public schemes and insurance initiatives, coverage remains inadequate and uneven, leaving a substantial share of older adults financially unprotected. These findings point to an urgent need to expand and strengthen publicly financed health insurance and financial risk protection, with targeted safeguards for households with older adults, to prevent healthcare costs from exacerbating poverty.

## 1 Introduction

India stands at a critical juncture of demographic and epidemiological transition, with the population aged 60 years and above projected to reach 319 million by 2050, comprising over 20% of the total population (23). While longer lifespans reflect improvements in public health and development, they also expose households to significant economic vulnerabilities, especially related to healthcare costs in the absence of comprehensive social protection. The burden of non-communicable diseases (NCDs) such as hypertension, diabetes, and cardiovascular conditions is disproportionately high among the older population. The National Sample Survey (NSS) 75th round (2017–18) reports that older adults individuals account for 24% of hospitalizations in India, despite constituting less than 9% of the population. Furthermore, over 70% of health expenditure among the older adults is paid out-of-pocket (OOP), with minimal insurance coverage. These OOP expenditures often result in catastrophic health spending, pushing vulnerable households into poverty.

According to the World Bank’s Poverty and Inequality Platform (2021) “11.09% of India’s population lived below the international poverty line of $2.15/day (PPP 2017) in 2018”. However, when accounting for health-related OOP expenses, the poverty headcount among older adults-headed households is significantly higher, indicating the impoverishing impact of medical costs. Previous studies have shown that catastrophic health expenditure (defined as health spending exceeding 10% or 25% of total household consumption) disproportionately affects households with older adults, especially in rural and lower-income strata (1, 2, 13).

Ageing population brings with it a complex set of socio-economic and healthcare challenges. The burden of out-of-pocket health expenditure (OOPHE) in India is among the highest globally, accounting for over 50% of total health spending (24). Households with older adults are especially vulnerable, as they often have limited income-generating capacity, depleted savings, and higher healthcare utilization (15, 20). These expenditures can quickly erode household consumption capacity, forcing families to reduce spending on essentials such as food, housing, and education, or to fall below the poverty line altogether. Despite this, the intersection of ageing, healthcare costs, and poverty remains underexplored in the Indian context. While there is growing literature on older adults wellbeing and health insurance, few studies have attempted to quantify the extent to which health expenditures push households with older adults into poverty (14, 16, 19). Most national poverty estimates do not adjust for post-medical expenditure consumption, thereby masking the financial distress caused by health shocks.

This study seeks to fill this empirical gap by examining the impact of health-related spending on poverty among households with older adults in India. By measuring pre- and post-health-expenditure poverty, we aim to uncover the hidden cost of ageing, a cost that is economic as much as it is social. The findings have significant implications for the design of financial protection schemes, older adults welfare programs, and the future of social security policy in an ageing India. The literature exists in analyzing the impact of OOPHE on poverty in India (3). Yet, there is limited literature that quantifies the direct impact of health spending on poverty transitions among older adults populations in India. This study addresses that gap by analyzing how health expenditure contributes to poverty among households with older adults using data from the NSS 75th round (2017–18) consumer expenditure survey.

This study aims to assess the impact of out-of-pocket (OOP) health expenditure on poverty, and to identify the key factors that influence such expenditures. In essence, the research is guided by the following objectives:

1. To examine the relationship between out-of-pocket health expenditure and the incidence of poverty.

2. To identify the determinants of poverty among households following OOP healthcare payments.The most recent national survey by National Sample Survey Organisation (2017–2018) has been used to extract household-level data for analyzing the impact of OOP expenditure on older adults household poverty.

## 2 Data source

This study utilizes unit-level data from the 75th round of the National Sample Survey (NSS) on Household Social Consumption: Health, conducted between July 2017 and June 2018 by the Ministry of Statistics and Programme Implementation (MoSPI), Government of India. The dataset includes information on key socio-economic characteristics such as household size, education, caste, income quintile, insurance coverage, and use of public or private healthcare facilities. The survey followed a stratified multi-stage sampling design, with villages (rural) and Urban Frame Survey blocks (urban) as first-stage units and households as second-stage units, selected via circular systematic sampling. For the purpose of this study, we focus on a nationally representative sub-sample of 31,194 households where at least one member is aged 60 years or above, capturing the health and economic vulnerability of households with older adults. It comprises 17,253 rural households and 13,941 urban households. In the absence of an officially updated poverty line for the 2017–18 period, this study adopts the Tendulkar Committee’s state-specific poverty lines, which are widely accepted and used in Indian poverty studies. These thresholds are adjusted for inflation to align with 2017–18 price levels, ensuring temporal and spatial comparability across states.

## 3 Methodology

To assess the impoverishing effects of health-related out-of-pocket expenditure (OOPHE) among households with at least one older adults member, we employ two standard poverty measures: the poverty headcount ratio and the poverty gap index. These indicators not only quantify the number of individuals pushed below the poverty line due to healthcare payments, but also capture the depth of poverty experienced by the already poor households.

Monthly *Per Capita* Expenditure (MPCE is used as a proxy for household income for calculation of poverty. The Out-of-pocket health expenditure (OOPHE) component comprises payments toward medical advice, package fees, doctor’s/surgeon’s fees, medicines, diagnostic tests, bed charges, and patient transportation, and is adjusted to monthly basis before being deducted from MPCE. To determine post-OOPHE poverty status, this adjusted OOPHE is subtracted from household income (MPCE), and the resulting figure is compared against the inflation-adjusted, state-specific poverty line derived using the Tendulkar Committee methodology. If income net of OOPHE remains above the relevant state poverty line, the household is classified as not poor; if it falls at or below the poverty line, the household is classified as poor. This approach follows the standard practice in the catastrophic health expenditure literature (1, 4).

The pre-OOPHE poverty headcount ratio (Pre-Hp) is computed by comparing each individual’s per capita consumption expenditure with the respective poverty line, as shown in Equation 1:


$$\mathrm{Pre}\;\mathrm{Hp}=\frac{1}{\mathrm{n\Sigma}}\;(x_{i}\leq \mathrm{PL})$$ (1)

Where $x_{i}$ is the per capita consumption expenditure of an individual (in Rupees), PL is the poverty line (in Rupees), n is the total number of individuals. This post-payment poverty headcount ratio (Post Hp) captures the additional burden of healthcare payments and is expressed in Equation 2:


$$\text{Post}\;\mathrm{Hp}=\frac{1}{\mathrm{n\Sigma}}\;((x_{i}-\text{OOPE})\leq \mathrm{PL})$$ (2)

To capture the impact of OOPHE, we compute the post-payment poverty headcount ratio (Post-Hp) by deducting household health expenditures from the total consumption, as given in Equation 3:


NewHp=PostHp–PreHp (3)

The difference between *Post-Hp* and *Pre-Hp* provides the share of individuals newly falling into poverty due to healthcare spending.

To assess the depth of poverty, we calculate the poverty gap, which reflects how far below the poverty line poor individuals fall, on average. The gap index is defined in Equation 4:


$$\text{Poverty}\;\mathrm{Gap}=\frac{1}{n}\Sigma \;(\mathrm{PL}-x_{i})\;\text{for}\;x_{i}\leq \mathrm{PL}$$ (4)

This index represents the average shortfall in consumption from the poverty threshold among the poor.

### 3.1 Multivariate (logit) modeling and variables description

To examine the determinants contributing to a household’s descent into poverty as a result of out-of-pocket healthcare spending, this study employs a logit regression framework. The model aims to identify factors that significantly influence the probability of a household becoming impoverished due to medical expenses. The change in average poverty levels before and after accounting for health-related OOPHE is taken as the effect of such expenditures on poverty outcomes. To assess these relationships more precisely, marginal effects derived from the logit model are reported. These reflect the incremental change in the probability of poverty associated with a one-unit change in each independent variable, holding all other factors constant.

A binary dependent variable is constructed to represent whether or not a household falls below the poverty line due to OOPHE. Households that remain above the poverty line after incurring health expenses are coded as ‘0’, while those pushed below the threshold are coded as “1”. This binary outcome not only serves as the dependent variable in the regression analysis but also represents the incidence of OOPHE-induced impoverishment among households.

The impoverishing effect of out-of-pocket health expenditure is well documented to vary systematically with the demographic and socio-economic profile of the household. Existing literature establishes that household-level characteristics such as household size, the presence of older adults or dependent members, and place of residence shape the extent to which health spending pushes a household below the poverty line, since larger households face proportionately greater consumption needs while their capacity to absorb a health shock is simultaneously stretched thinner across members (1, 2). Similarly, for any given level of health expenditure, the depth of its impoverishing effect is conditioned by the household’s underlying economic cushion, which is itself a function of the household head’s educational attainment (12, 17) and the broader socio-economic status of the household, including caste-based structural disadvantage that continues to shape access to income, assets, and formal employment in the Indian context (5). Health insurance coverage, in turn, is consistently found (22) to lower the probability of catastrophic and impoverishing health spending by pooling risk across members, whereas the type and duration of care sought, particularly reliance on costlier private facilities and prolonged hospitalization episodes, tend to increase the financial burden borne directly by the household (6, 7, 16, 19).

Building on this literature, the present study measures the socio-economic and demographic profile of older adults-inclusive households using household size (categorized as small, medium, and large), caste category (General, OBC, SC, and ST), and the highest level of education attained within the household, ranging from no literate member to graduate and above. Health-seeking behavior is captured through whether the household relied on private or public healthcare facilities, along with the duration of hospitalization, grouped into short (under 5 days), medium (5–20 days), and prolonged (20 days and above) stays, given the established gradient between length of hospitalization and the magnitude of out-of-pocket cost. Health insurance coverage is included as a binary indicator of whether at least one household member was insured, reflecting its protective role against catastrophic spending. Finally, the natural log of out-of-pocket health expenditure is included to capture the direct financial magnitude of the health shock itself, consistent with the approach of quantifying the incremental effect of health spending intensity on the probability of a household’s descent into poverty.

## 4 The burden of healing: medical costs and the descent into poverty

Table 1 shows the prevalence of the poverty among households where at least one member is aged, i.e., age equal to or above 60 years. Using state-specific poverty thresholds based on the Tendulkar methodology for Monthly *Per Capita* Expenditure (MPCE), adjusted for inflation, the estimated pre-OOPHE poverty rates among households with at least one older adults member are 20.45% in rural areas and 6.86% in urban areas. At the aggregate level, the poverty rate is 13.66%. The older adults population faces higher likelihood for illness. They frequently require medical attention, leading to recurrent and often substantial OOPHE. This continuous expenditure erodes household income, forcing many above the poverty line into a state of destitution. This study seeks to assess the impact of out-of-pocket health expenditure (OOPHE) on health among households with at least one older adults member. After deducting inflation-adjusted OOPHE on health from household income, poverty rates rise to 22.43% in rural areas, 9.84% in urban areas, and 16.14% at the national level—reflecting respective increases of 1.98, 2.98, and 2.48%. This overall increase corresponds to approximately 1.93 million additional households falling below the poverty line.

**Table 1:** Prevalence of poverty.

Sector	Pre-OOPHE poverty rate (%)	Post-OOPHE poverty rate (%)	Total difference (%)
Rural	20.45	22.43	1.98
Urban	6.86	9.84	2.98
Overall	13.66	16.14	2.48

Source: Authors’ calculation from the NSS 75th Round: Household Social Consumption – Health 2017–2018.

The rise in poverty is more pronounced in urban areas. Although poverty remains more prevalent in rural regions, increased migration to urban centers has not translated into improved living conditions due to limited employment opportunities. This reflects the Harris-Todaro model (8) which predicts that rural workers move to cities expecting higher wages, but end up in low-paying informal jobs or unemployment. When adult children face unemployment or low earnings in cities, older adults parents often send money or work longer, which reduces their own wellbeing and increases financial stress (9) Using a nowcasting approach, (10) estimated that 7.2% of India’s urban population lived in extreme poverty in 2017, underscoring the continued vulnerability of many migrants in urban labor markets. Consequently, low-income urban workers remain particularly vulnerable to health shocks, with rising OOPHE contributing to the growing trend of urban poverty (4). Another contributing factor may be the relatively higher cost of treatment in private hospitals in urban areas compared to rural regions. This makes older adults individuals in urban settings more susceptible to incurring elevated out-of-pocket health expenditures, thereby increasing their likelihood of falling into poverty (5).

## 5 Results

The descriptive statistics in Table 2, based on a sample of 31,194 households with older adults, reveals important insights into the household structure, health conditions, healthcare utilization, education, income distribution, and the economic burden of healthcare. In terms of household size, medium-sized families (with 5 to 8 members) constitute the majority, accounting for 54.79% of the total sample, while small households (with four or fewer members) and large households (with nine or more members) make up 32.70 and 12.51%, respectively. This suggests that joint or extended family systems are still prevalent in a significant portion of Indian households.

**Table 2:** Descriptive statistics.

Variable	Definition	Frequency *N* = 31,194	Percentage %
Household size	Small household (≤4)	10,201	32.70
	Medium household- (5–8)	17,091	54.79
	Large household- (≥9)	3,902	12.51
Chronic illness	Inclusion of at least one individual with chronic ailment in last 365 days	9,721	31.16
Caste	Others	11,517	36.92
	ST	4,596	14.73
	SC	2,900	9.30
	OBC	12,181	39.05
Health insurance coverage	At least one member of the household is covered by the insurance	7,476	23.97
Duration of stay in hospital	≤5 days of stay	5,060	16.22
	6–10 days of stay	3,869	12.40
	11–20 days of stay	1,786	5.73
	≥20 days of stay	914	2.93
Usage of a private healthcare facility	Privately maintained medical facilities providing healthcare services in exchange for money.	11,990	38.44
General education	Not literate	7,280	23.34
	Primary education	10,015	32.11
	Secondary education	5,215	16.72
	Higher secondary	3,822	12.25
	Graduate and above	4,862	15.59
Income quantile	Poorest income quintile (reference)	4,677	14.99
	Second income quintile	4,937	15.83
	Third income quintile	5,503	17.64
	Fourth income quintile	7,090	22.73
	Highest income quintile	8,987	28.81

Variables	Definition and categories	Mean	Std. error
Poverty gap:	The ratio by which the mean income of the poor falls below the poverty line		
	Rural	0.18	0.005
	Urban	0.189	0.013
	Overall	0.181	0.005
Reimbursement ratio	Total amount reimbursed/Total medical expenditure		
	Rural	0.027	0.003
	Urban	0.083	0.005
	Overall	0.046	0.002
Out of pocket expenditure	Average out of pocket expenditure by household on medical (in Rs.)		
	Rural	20,997	966
	Urban	39,884	1,481
	Overall	27,495	816

Source: Authors’ calculation from the NSS 75th Round: Household Social Consumption – Health 2017–2018.

Health-related indicators show that 31.16% of households reported at least one member suffering from a chronic illness in the preceding 365 days, indicating a substantial burden of long-term health conditions. Despite this, health insurance coverage remains alarmingly low, with only 23.97% of households reporting that at least one member was insured. This lack of coverage makes households vulnerable to financial shocks resulting from medical expenses.

Hospitalization data further supports this concern. Most hospital stays were relatively short in duration, with 16.22% of hospitalizations lasting up to 5 days, and progressively fewer cases as the length of stay increased—12.40% for 6–10 days, 5.73% for 11–20 days, and just 2.93% for stays beyond 20 days. However, even short-term hospitalizations can result in significant expenditures, especially when private healthcare is used. Notably, 38.44% of households reported utilizing private healthcare services, which typically incur higher costs than public alternatives. This reliance on private providers could be due to perceptions of better quality or accessibility in private facilities, especially in urban areas.

The educational profile of households shows that 23.34% have no literate member, highlighting continued gaps in basic education. Nevertheless, a majority of households have members who have completed at least primary education (32.11%), with others attaining secondary (16.72%), higher secondary (12.25%), or graduate and above (15.59%) levels. Education is a critical determinant of health awareness, access to better employment, and financial resilience, which directly affects how households manage health-related expenses.

Income quantile data indicates a relatively skewed distribution toward higher income brackets, with the richest quintile comprising 28.81% of the sample and the poorest, 14.99%.

Further, the analysis of economic vulnerability due to health expenditures presents concerning trends. The poverty gap, which measures how far below the poverty line the average poor household falls, stands at 0.180 in rural areas and 0.189 in urban areas, with an overall gap of 0.181. This suggests that on an average, poor households earn about 18% less than the threshold income required to meet basic needs. Financial protection against medical expenses is extremely limited, as shown by the reimbursement ratio—only 2.7% of total medical expenditure is reimbursed in rural households and 8.3% in urban ones, with a national average of 4.6%. This reflects the inadequacy of insurance mechanisms and public support systems in shielding households from medical shocks.

The burden of out-of-pocket health expenditure (OOPHE) is particularly striking. Urban households spend an average of ₹39,884 on medical care, nearly double the rural average of ₹20,997. The overall average OOPHE is ₹27,495, underscoring the significant financial strain that medical needs impose on households, particularly in the absence of adequate insurance coverage. The higher OOPHE in urban areas could be attributed to greater reliance on expensive private healthcare, coupled with a higher prevalence of non-communicable diseases among the urban older adults population.

### 5.1 Multivariate regression analysis results

In Table 3, the regression model investigates the likelihood of a household falling below the poverty line due to health-related out-of-pocket expenditure (OOPHE). The marginal effects provide insights into how various household characteristics influence this probability. Household size emerges as a significant predictor. Compared to small households (≤4 members, reference category), medium-sized households (5–8 members) have a probability that is 6.4 percentage points higher of falling into poverty due to health spending, while large households (≥9 members) show a 16.4 percentage point increase. This indicates that larger households face greater financial vulnerability, likely due to increased consumption needs and potential health burden spread across more members. Use of private healthcare is significantly associated with higher poverty risk. Households that sought treatment in private hospitals have a probability 2.1 percentage points higher of falling below the poverty line compared to those relying on public facilities, suggesting the higher cost burden associated with private care. Caste also plays a critical role. Compared to households from the General category, Scheduled Caste (SC) and Scheduled Tribe (ST) households have a probability 13.2 and 21.1 percentage points higher of falling into poverty, respectively, while Other Backward Classes (OBC) face a 6.9 percentage point increase. These findings point to persistent socio-economic disparities and structural disadvantages among marginalized groups.

**Table 3:** Logit regression results.

Variables	Category	Marginal effects	Std. error	*p*
Household size	Small household (reference)			
	Medium household	0.064	0.010	0.000
	Large household	0.164	0.020	0.000
Caste	Others (reference)			
	SC	0.132	0.016	0.000
	ST	0.211	0.024	0.000
	OBC	0.069	0.010	0.000
Education	Below primary (reference)			
	Primary	−0.030	0.014	0.029
	Secondary	−0.084	0.015	0.000
	Higher secondary	−0.079	0.017	0.000
	Graduate and above	−0.139	0.014	0.000
Duration of stay in hospital	Not staying in hospital(reference)			
	Less than 5 days	−0.021	0.010	0.040
	5–10 days of stay	0.043	0.017	0.014
	11–20 days of stay	0.134	0.021	0.000
	20 and above days of stay	0.221	0.035	0.000
Usage of a private healthcare facility:	Admitted to private hospital	0.021	0.010	0.034
Out of pocket health expenditure	Log of OOPHE on health	0.014	0.001	0.000
Health insurance coverage:	At least one is covered under insurance	−0.081	0.012	0.000
Constant	constant	−1.536	0.072	0.000

Source: Authors’ calculation from the NSS 75th Round: Household Social Consumption – Health 2017–2018.

*N* = 31,194; Wald *χ*^2^(16) = 784.34, *p* < 0.001; Log pseudolikelihood = −25,063,759; Pseudo *R*^2^ = 0.1024.

The log of OOPHE on health has a statistically significant positive effect; a 1% increase in OOPHE is associated with an approximately 0.014 percentage point increase in the probability of falling into poverty. It underlines the direct financial threat posed by medical expenses. Educational attainment within the household is inversely related to the probability of impoverishment. Relative to households where the highest education level is below primary, the probability of falling into poverty is lower by 3.0 percentage points for primary education, 8.4 percentage points for secondary, 7.9 percentage points for higher secondary, and 13.9 percentage points for graduate and above. This trend suggests that higher education equips individuals with better income opportunities and access to information, reducing the financial shock of health expenditures.

Health insurance coverage has a protective effect, with households having at least one insured member having a probability 8.1 percentage points lower of falling into poverty. This underscores the role of risk pooling mechanisms in mitigating catastrophic health spending. The duration of hospital stay also shows a clear gradient in risk. Compared to households with no hospitalization, short hospital stays (<5 days) associated with a 2.1 percentage point lower poverty risk, possibly due to its minimal cost or elective nature. However, hospital stays of 5–10 days, 11–20 days, and ≥20 days are associated with a 4.3, 13.4, and 22.1 percentage point higher probability of poverty, respectively. This escalating trend emphasizes the burden of prolonged treatment durations on household finances.

## 6 Conclusions and discussion

This study provides robust evidence on the impoverishing effects of out-of-pocket health expenditure (OOPHE) on health among Indian households with at least one older adults member. The findings demonstrate a notable rise in poverty levels once healthcare-related expenses are deducted from household income, indicating the significant economic vulnerability imposed by medical costs. This burden is particularly acute in urban areas, where both the average OOPHE and the reliance on private healthcare facilities are substantially higher. Despite relatively lower poverty rates in urban areas compared to rural regions, the increase in poverty due to health spending is more pronounced in cities, underscoring the inadequacy of current health protection mechanisms for older adults populations.

The analysis reveals systemic gaps in health insurance coverage, limited financial protection, and persistent socio-economic disparities. A substantial proportion of households reported chronic illness and hospitalization, yet the reimbursement for health expenses remains alarmingly low. The study also points to the continued existence of joint and extended family systems, yet these are not sufficient to buffer the financial shocks associated with prolonged illness or hospitalization. Furthermore, low levels of education and unequal income distribution compound the challenges faced by households with older adults in managing health expenditures (21).

Our findings are in line with those of earlier studies (such as (1, 2); and (6)) and mention a few key findings which are in line with the existing studies (11, 18, 22). Consistent with Tripathy et al. (2), we find that longer hospital stays and reliance on private healthcare facilities substantially heighten the risk of OOPE-induced impoverishment among households with older adults, This study advances existing research by using the most recent nationally representative data (NSS 75th Round) restricted to older adults-inclusive households, and by jointly estimating poverty headcount, poverty gap, and impoverishment determinants within a single framework.

These findings are situated within the broader context of India’s demographic shift, with the older adults population projected to double in the next two decades. This transition poses critical challenges to healthcare financing, service delivery, and social protection systems. While initiatives such as Ayushman Bharat, PMJAY, and the National Programme for Health Care of the Elderly (NPHCE) are steps in the right direction, their reach and budgetary allocations remain limited in scope and effectiveness. Recent estimates suggest that less than one-fourth of households with older adults have any form of insurance coverage, and public expenditure on older adults healthcare continues to be modest despite rising need.

As India navigates this demographic transformation, addressing the health-related economic vulnerability of households with older adults must become a policy priority. This study underscores the importance of expanding financial risk protection through better insurance penetration, increased public investment in geriatric care, and targeted interventions for vulnerable groups such as Scheduled Castes, Scheduled Tribes, and low-income urban migrants. Moreover, bridging educational and income inequalities can further strengthen households’ resilience to medical shocks.

Our study has certain unique strengths, such as its large, nationally representative sample of older adults-inclusive households drawn from the NSS 75th Round, sub-group analysis across rural, urban, and caste categories, and its use of a rural–urban disaggregated, state-adjusted poverty line based on the Tendulkar methodology, Nonetheless, the study has limitations. Its reliance on cross-sectional survey data restricts causal inference and temporal analysis. The measurement of poverty is confined to consumption-based thresholds, omitting multidimensional aspects of wellbeing such as social inclusion, care availability, and psychological distress. Additionally, the self-reported nature of health expenditure data may be subject to recall bias. Future research should adopt longitudinal and mixed-methods approaches to capture the evolving nature of older adults poverty and assess the long-term effectiveness of policy interventions. Moreover, the prevalence of poverty is likely to get affected by the choice of the poverty line. Future research should adopt longitudinal and mixed-methods approaches to capture the evolving nature of older adults poverty and assess the long-term effectiveness of policy interventions.

In sum, this study contributes to the growing body of literature highlighting the intersection of ageing, health, and poverty. It calls for a renewed policy focus on the financial protection of households with older adults to ensure that ageing in India is not synonymous with impoverishment, but rather with dignity, stability, and security.

## Data Availability

The original contributions presented in the study are included in the article/supplementary material, further inquiries can be directed to the corresponding author/s.

## References

[1] Berman P Ahuja R Bhandari L. The impoverishing effect of healthcare payments in India: new methodology and findings. Econ Polit Wkly. (2010) 45:65–71.

[2] Prince DEA Kodali PB. Financial impact of non-communicable diseases on households with older adults in india: a mixed methods study. BMC Geriatr. (2026) 26:772. doi: 10.1186/s12877-026-07493-9 PMC13224535 42000994

[3] Soni N Nayak RJ Blon P Ghosh M Singh K Singh A. Impact of out-of-pocket health expenditure on poverty in India. Front Public Health. (2026) 14:1762886. doi: 10.3389/fpubh.2026.1762886, 41938958 PMC13044129

[4] Sriram S Albadrani M. Impoverishing effects of out-of-pocket healthcare expenditures in India. J Family Med Prim Care. (2022) 11:7120–8. doi: 10.4103/jfmpc.jfmpc_590_22, 36993034 PMC10041239

[5] Vasudevan U Akkilagunta S Kar SS. Household out-of-pocket expenditure on health care—a cross-sectional study among urban and rural households, Puducherry. J Family Med Prim Care. (2019) 8:2278–82. doi: 10.4103/jfmpc.jfmpc_302_19, 31463242 PMC6691471

[6] Ahmad F Mohanty PC. Incidence and intensity of catastrophic health expenditure and impoverishment among the elderly: an empirical evidence from India. Sci Rep. (2024) 14:15908. doi: 10.1038/s41598-024-55142-1, 38987298 PMC11237111

[7] Kumar K Singh A Kumar S Ram F Singh A Ram U . Socio-economic differentials in impoverishment effects of out-of-pocket health expenditure in China and India: evidence from WHO SAGE. PLoS One. (2015) 10:e0135051. doi: 10.1371/journal.pone.0135051, 26270049 PMC4536075

[8] Harris JR Todaro MP. Migration, unemployment and development: a two-sector analysis. Am Econ Rev. (1970) 60:27–43. doi: 10.4337/9781035362813.00010

[9] Zhang C Lyu K Cheng X Zhang C. The impact of adult children rural–urban migration on left-behind parents' health: evidence from China. Front Public Health. (2022) 10:951124. doi: 10.3389/fpubh.2022.951124, 36203690 PMC9530906

[10] Edochie IN Freije-Rodriguez S Lakner C Moreno Herrera L Newhouse DL Sinha Roy S . What Do We Know about Poverty in India in 2017/18? World Bank Policy Research Working Paper No. 9931. Washington, DC: World Bank (2022).

[11] Arsenijevic J Pavlova M Rechel B Groot W. Catastrophic health care expenditure among older people with chronic diseases in 15 European countries. PLoS One. (2016) 11:1–18. doi: 10.1371/journal.pone.0157765, 27379926 PMC4933384

[12] Azzani M Roslani AC Su TT. Determinants of household catastrophic health expenditure: a systematic review. Malays J Med Sci. (2019) 26:15–43. doi: 10.21315/mjms2019.26.1.3, 30914891 PMC6419871

[13] Berki SE. A look at catastrophic medical expenses and the poor. Health Aff. (1986) 5:138–45. doi: 10.1377/hlthaff.5.4.138, 3102333

[14] Brinda EM Kowal P Attermann J Enemark U. Health service use, out-of-pocket payments and catastrophic health expenditure among older people in India: the WHO study on global ageing and adult health (SAGE). J Epidemiol Community Health. (2015) 69:489–94. doi: 10.1136/jech-2014-204960, 25576563

[15] Grundy E. Ageing and vulnerable elderly people: European perspectives. Ageing Soc. (2006) 26:105–34. doi: 10.1017/S0144686X05004484

[16] Kastor A Mohanty SK. Disease-specific out-of-pocket and catastrophic health expenditure on hospitalization in India: do Indian households face distress health financing? PLoS One. (2018) 13:e0196106–18. doi: 10.1371/journal.pone.0196106, 29746481 PMC5945043

[17] Khan JAM Ahmed S Evans TG. Catastrophic healthcare expenditure and poverty related to out-of-pocket payments for healthcare in Bangladesh-an estimation of financial risk protection of universal health coverage. Health Policy Plan. (2017) 32:1102–10. doi: 10.1093/heapol/czx048, 28575415

[18] Liu S Coyte PC Fu M Zhang Q. Measurement and determinants of catastrophic health expenditure among elderly households in China using longitudinal data from the CHARLS. Int J Equity Health. (2021) 20:62–9. doi: 10.1186/s12939-020-01336-8, 33608014 PMC7893946

[19] Panda BK Mohanty SK. Catastrophic health spending among older adults in India: role of multiple deprivation. Aging Health Res. (2022) 2:100100. doi: 10.1016/j.ahr.2022.100100

[20] Pandey P Singh S. Catastrophic health expenditure among geriatric population of Lucknow district, India. J Geriatr Ment Health. (2017) 4:42. doi: 10.4103/jgmh.jgmh_40_16

[21] Srivastava S Chauhan S Patel R. Socio-economic inequalities in the prevalence of poor self-rated health among older adults in India from 2004 to 2014: a decomposition analysis. Ageing Int. (2021) 46:182–99. doi: 10.1007/s12126-020-09385-8

[22] Zhou Y Wushouer H Vuillermin D Guan X Shi L. Does the universal medical insurance system reduce catastrophic health expenditure among middle-aged and elderly households in China? A longitudinal analysis. Eur J Health Econ. (2021) 22:463–71. doi: 10.1007/s10198-021-01267-3, 33582893

[23] United Nations DESA. DESA, Population Division. World Population Prospects 2022: Summary of Results. New York: UN (2022).

[24] Ministry of Health and Family Welfare, Government of India. National Health Accounts Estimates for India. New Delhi: NHSRC (2021).

